# Appearances Are Deceptive: Two Case Reports in Teenagers on the Conservative Laparoscopic Surgery for Adnexal Torsion with Apparent Infarction

**DOI:** 10.1155/2022/1798599

**Published:** 2022-04-27

**Authors:** A. Jayawardane, M. Patabendige, C. Gunathilaka

**Affiliations:** ^1^Obstetrics and Gynaecology, Faculty of Medicine, University of Colombo, Colombo, Sri Lanka; ^2^Obstetrician and Gynaecologist, Base Hospital, Mahaoya, Sri Lanka; ^3^National Hospital of Sri Lanka, Colombo, Sri Lanka

## Abstract

*Introduction*. Torsion of the ovary, tube, or both is estimated to be responsible for 2-7% of all gynaecological emergencies. Oophorectomy is commonly performed for adnexal torsion with a possible negative impact on fertility in women of reproductive age. *Case Presentations*. We report two cases of teenage girls presented with adnexal torsion describing their laparoscopy features. Detorsion without additional surgical intervention could save their ovaries. *Discussion*. Detorsion is a more conservative surgical approach that should be considered in all younger women with ovarian torsion.

## 1. Introduction

Ovarian torsion refers to the full or partial twisting of the ovary around its suspensory structures, leading to the compromised blood supply to the ovary. The resultant ischemia can cause tissue necrosis with a potential loss of the affected ovary permanently. Oophorectomy was the standard treatment for many decades due to the presumed risk of thrombosis, infection, peritonitis, and reperfusion injury following untwisting of the twisted ovary [1]. The presence of adnexal cysts may result in torsion of the adnexa, with a similar mechanism leading to ischaemia and necrosis. It is estimated that 2.7% to 7.4% of all gynaecological emergencies are due to torsion [2] [3].

This paper summarises similar case reports of two, young girls presenting with adnexal torsion where the visual inspection was highly suggestive of nonviable ischaemic necrosis of the tissues. However, proceeding to conservative laparoscopic surgery resulted in a successful outcome.

## 2. Case Presentations

### 2.1. Case: 1

A 15-year-old, previously healthy, Sri Lankan, Muslim ethnic girl presented with severe lower abdominal pain of two days duration. The pain was gradually getting worse. Her menarche was four years ago, and she had regular periods with no dysmenorrhoea. On direct inquiry, she has had three similar episodes during the last six months, but less severe in intensity. On examination, the abdomen was tender, mainly in the left iliac fossa. Urine human chorionic gonadotrophin was negative. Ultrasound revealed a 12 cm complex pelvic mass. The overall picture was suggestive of a twisted ovarian cyst with features of surrounding oedema.

She underwent laparoscopy with consent for the removal of the affected ovary. At laparoscopy, an adnexal twisting due to a left ovarian cyst was confirmed. The left tube was grossly edematous. Both ovary and the tube appeared congested, dark blue-black in colour, and gross appearance suggestive of nonviable tissues. Adnexa was untwisted and ovarian cystectomy was done. At the end of surgery (one hour), significant improvement of appearance and perfusion was noted. Her recovery was uncomplicated with low molecular weight heparin and early mobilisation. The extracted cyst wall was compatible with a benign serous cystadenoma.

**Figure 1:**
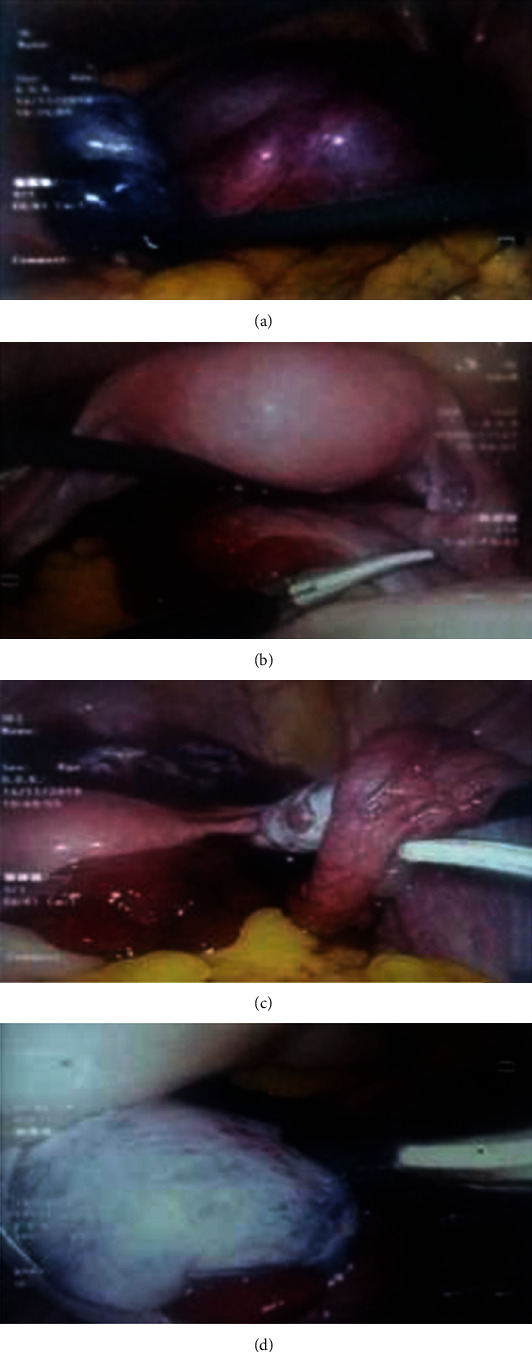
Sequential changes in the ovarian and tubal tissues in the teenager related to the case 1.

Three months later, an ultrasound scan on day-15 revealed a corpus luteum in the left ovary.  Figure 1 shows the sequential changes in the ovarian and tubal tissues.

### 2.2. Case: 2

A 13-year-old, previously healthy, Sri Lankan, Sinhala ethnic girl presented with worsening lower abdominal pain of 36 hours duration. Her menarche was one year ago. She was uncertain in her last period. On examination, she had severe right-sided lower abdominal tenderness. Urine human chorionic gonadotrophin was negative. An ultrasound scan revealed a large 6 cm complex right adnexal cyst with possible surrounding oedema.

**Figure 2:**
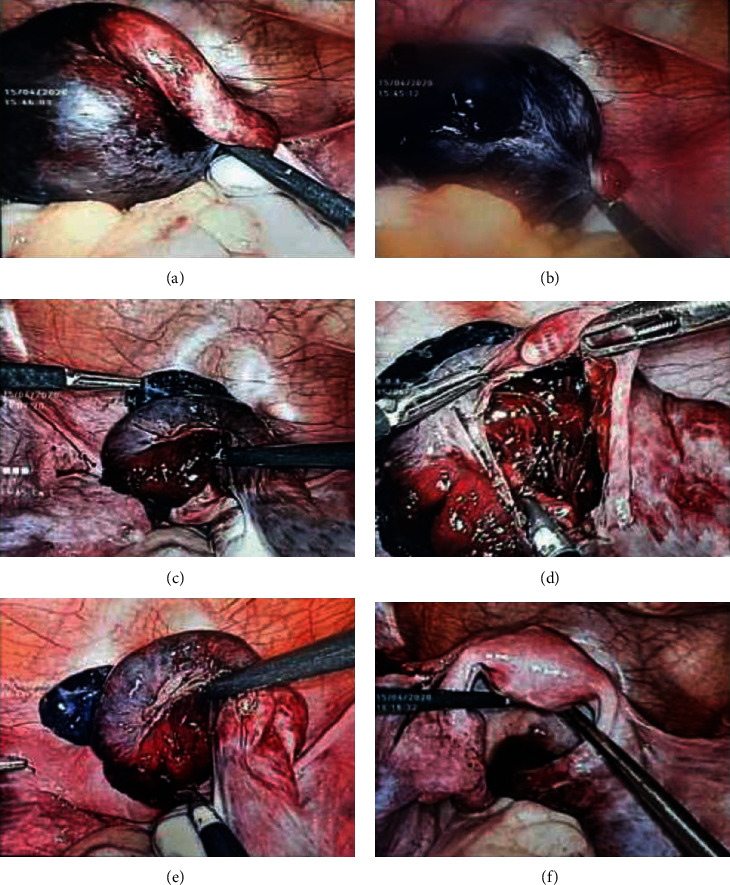
Sequential changes in the ovarian and tubal tissues in the teenager related to the case 2.

At laparoscopy, a twisted para-ovarian cyst and fallopian tube were noted. There was significant tissue edema and the fallopian tube distal to the torsion was dark blue-black in colour and appeared nonviable. Right ovary was not involved in the torsion. Left adnexa was normal. The fallopian tube was untwisted, and removal of cyst was carried out. At the end of surgery (45 minutes), significant reperfusion was noted in the fallopian tube. The patient had an uncomplicated recovery. Cyst wall histology revealed a benign serous cyst with foci of haemorrhage.  Figure 2 shows the sequential changes in the right fallopian tube.

## 3. Discussion

### 3.1. Pathophysiology of Adnexal Torsion

Adnexal torsion is an uncommon gynaecological emergency presenting with worsening lower abdominal/pelvic pain [4]. Some women describe previous self-limiting similar pain episodes, usually attributed to partial torsion followed by spontaneous de-torsion. Persistent torsion leads to tissue congestion due to impeded venous drainage followed by ischaemia as arterial blood supply is compromised leading to tissue necrosis. Early identification and intervention are important to prevent permanent tissue damage and toxaemia.

### 3.2. Challenges in Diagnosis

In our experience, high-resolution transvaginal ultrasound (used trans-rectally if the patient is before coitarche) is an extremely useful tool available at the clinic setting. We use colour Doppler to assess limitations on the blood flow due to torsion. We aim for a presentation-to-surgery time of less than 6 hours, which is usually feasible. Diagnosis is challenging but early diagnosis and treatment can prevent catastrophic complications [5]. Although Doppler may reveal limitations on blood flow, there are no definitive tests to confirm tissue viability before surgery. Traditionally, one depends on a visual assessment of viability by the surgeon for decision making.

Laparoscopic cystectomy during torsion in a case of complex cyst might pose a potential concern due to possible oncologic problems. However, the age-adjusted incidence of ovarian malignancy is very rare, 1.072 per 100,000 per year in girls aged 10–19 years [6]. In addition, we are applying the International Ovarian Tumor Analysis (IOTA) classification (B rules and M rules) in every ovarian mass which has a higher sensitivity and specificity to rule out a malignant one [7]. There was no indication to go for magnetic resonance imaging, and a postsurgical histology report was sought as early as possible.

### 3.3. Surgical Management

Preexisting adnexal cysts and ovarian hyperstimulation are considered to increase the risk of torsion. Torsion may happen in all age groups, but is more common in the younger, reproductive-age females [4]. Complications attributed to torsion and tissue necrosis include increased risk of thrombosis, infection, peritonitis, and reperfusion injury [4]. Removal of the affected adnexa was the traditional standard practice for several decades worldwide [1, 8]. However, oophorectomy in a young woman carries significant implications for future fertility. Despite some reports of successful cases of conservative management, surgical management was the norm for the past few decades [1, 8–10]. The concerns of this mode of treatment are fertility and loss of ovarian tissue at a younger age [11]. Recent two reviews on adnexal torsion have emphasized the importance of conservative management among adolescents [4, 11].

### 3.4. Conservative Management

As the edematous, ischemic, distorted adnexal tissue is extremely friable, laparoscopic detorsion is a delicate, time-consuming process. The slow process is likely to be beneficial as gradual detorsion is thought to be protective in reducing reperfusion injury of the ischaemic adnexa [12]. Most of these girls have benign tumours hence conservative management is a good option [8]. A recent consensus on conservative management has arisen giving rise to promising results [13–15]. A recent large population-based study of around 20,000 ovarian torsions demonstrated that conservative management may not be associated with increased perioperative complications, and the application of conservative options has increased from 18.9% to 25.1% between 2001 and 2015 [14].

The present account consisting of images provides a novel insight and supports conservative management. Both patients recovered with no peri-operative and postoperative complications. An immediate change in colour was obvious at laparoscopy in both cases. There is a paucity of robust data on the long-term follow-up of conservatively managed adnexal torsion. However, some recent reports showed that long-term outcomes were good [9, 14]. Case: 2 was followed up until three months and revealed a corpus luteum indicating complete recovery of the affected ovary. In case 2, there were no complaints at 6 months follow-up postsurgery. Therefore, these two teenage girls benefited from conservative treatment rather than radical surgery even when the adnexa appeared to be necrotic [5].

A study discussing long-term follow-up experience of conservative management has mentioned successfully managed two similar cases. The first patient has normally delivered her baby, and the other one is in her seventh month of pregnancy [16]. Another retrospective case-control study (*n* = 33) has compared laparoscopic surgical versus conservative organ-sparing management [17]. In this study, the mean age was 34.9 years (range 14-68 years) [17]. Out of 33, 17 cases had “conservative” management of which only two had only detorsion alone. Their objective was to evaluate the effectiveness of laparoscopic surgery for adnexal torsion reducing the need for a laparotomy. Therefore, short-term outcomes of conservative management have not been clearly mentioned.

### 3.5. Drawbacks of Conservative Management

Potential drawbacks of the conservative option are possibility of adhesion around the tube and ovary, tubal occlusion, and procedure-related complications such as laparoscopy [15]. One case reported in 1995 reported a case of conservative management of an ovarian torsion leading to adnexal necrosis in two days after detorsion [18]. A three-year follow-up study by Fujishita et al. has reported 14 cases of conservative management of adnexal torsion with a good outcome, and only two cases [2/14] have been reported having significant tubal adhesions leading to tubal occlusion in second-look laparoscopy [15]. The second case discussed here is at risk of developing such long-term complications.

## 4. Conclusion

Prompt intervention to preserve ovarian function should be laparoscopic wherever possible. Detorsion should be the treatment of choice in young girls and women of reproductive age whose families are not complete, regardless of the colour and appearance of the ovary at the time of surgery. The two cases presented could be an interesting starting point for larger studies on conservative treatment in young patients with adnexal torsion.

## Data Availability

No data to share. All the details have been mentioned in the manuscript.
